# Maternal Vitamin A Deficiency during Pregnancy and Its Relation with Maternal and Neonatal Hemoglobin Concentrations among Poor Egyptian Families

**DOI:** 10.1155/2013/652148

**Published:** 2013-08-21

**Authors:** A. M. Hamdy, M. M. Abdel Aleem, A. A. El-Shazly

**Affiliations:** ^1^Pediatrics Department, Faculty of Medicine, Ain Shams University, Cairo, Egypt; ^2^Gynecology and Obstetrics Department, Faculty of Medicine, Ain Shams University, Cairo, Egypt; ^3^Ophthalmology Department, Faculty of Medicine, Ain Shams University, Cairo, Egypt

## Abstract

*Background*. Vitamin A deficiency (VAD) during pregnancy represents a major public health problem in developing countries. Anemia is a common consequence of VAD. We aimed to measure serum retinol concentrations of a sample of poor Egyptian mothers and correlate it with their Hb% and cord Hb%. *Methods*. This cross-sectional study included 200 healthy mothers and their healthy full term newborns. Maternal and cord blood samples were collected for CBC and measurement of serum retinol concentrations. *Results*. Forty-seven mothers (23.5%) had VAD and 50% were anemic. Mothers with VAD had a significantly lower mean Hb% and a significantly higher frequency of anemia (95.7%) compared to mothers without VAD (35.9%). The relative risk for anemia among mothers with VAD was 2.7 (CI = 2.12–3.3). Newborns of mothers with VAD had a significantly lower mean cord Hb% compared to newborns of mothers without VAD. Maternal serum retinol concentrations were positively correlated with maternal Hb% and cord Hb%. *Conclusion*. Maternal VAD during pregnancy among poor mothers is associated with maternal anemia and lower Hb% of newborns at birth. Vitamin A supplementation is highly recommended for this vulnerable group.

## 1. Introduction

The requirements of vitamin A are increased during pregnancy. A daily intake of 800 *μ*g retinol equivalents was recommended as a safe level of vitamin A for pregnant women [1]. According to WHO, 7.8% of pregnant women in Africa have night blindness and 15.3% have low serum retinol concentrations [2]. Vitamin A deficiency (VAD) during pregnancy is associated with increased maternal mortality [3] and increased infant mortality rates during the first year of life [4].

According to WHO, anemia globally affects 41.8% of pregnant women all over the world and 57.1% of pregnant women in Africa [5]. Maternal anemia during pregnancy increases the maternal mortality [6] and has many adverse effects on fetal outcome including small for gestational age, premature rupture of membranes, preterm delivery. Severe maternal anemia may be associated with stillbirth and neonatal deaths [7]. 

Vitamin A is known to play a role in hematopoiesis, and anemia is a common consequence of VAD [8]. Vitamin A supplementation during pregnancy was found to improve maternal Hb% [9]. The aim of the present study was to measure serum retinol concentrations of a cohort of poor Egyptian mothers and correlate it with their hemoglobin concentrations (Hb%) and cord Hb% of their respective newborns.

## 2. Subjects and Methods

This cross-sectional study included 200 full term newborns and their respective mothers who were recruited from the reception room of a University Gynecology and Obstetrics Hospital, Cairo, Egypt, during the period from June 2011 to December 2011. This hospital provides antenatal and obstetric care for pregnant women at an Urban Metropolitan in Cairo of low socioeconomic standard. The study was approved by the local ethical committee of the Faculty of Medicine.

Online statistical calculator “http://www.raosoft.com/” was used for sample size calculation guided by confidence level of 95% and *α* error of 5%. The sample size was calculated to be 195 mother-infant pairs. 

We included only apparently healthy mothers, aged 19–39 years, with singleton pregnancy and proper spacing in-between pregnancy (a gap of more than 18 months from birth to subsequent conception) who delivered through uncomplicated spontaneous vaginal delivery. All included mothers were of low income (less than 53.43 US dollars per capita per months) [10]. We excluded all women with grand multiparity (more than 5 deliveries), complicated pregnancy including multiple pregnancy, established diagnosis of maternal anemia during pregnancy, preeclampsia, renal disease, antepartum hemorrhage (placental abruption placentae, placenta previa, vasa previa), history of fever, and signs of acute infection as well as mothers who had delivered through instrumental vaginal delivery or cesarean section. We also excluded all women who had vitamin A supplementation or history of exposure to teratogens.

We excluded neonates who were delivered before 37 weeks and had birth weight below 2500 grams. Neonates with congenital anomalies, birth trauma were also excluded as well as neonates with family history of hemolytic anemia or maternofetal incompatibility (positive Coombs' test or high reticulocyte count).

During the 6 months period, among 2058 deliveries 1554 mothers were excluded, 96 refused participation in the study, and 208 newborns were excluded. Causes of exclusion of mothers included inappropriate age for the study (87), grand multiparity (194), improper spacing in-between pregnancies (110), complicated pregnancy (204), exposure to teratogens (5), vitamin A supplementation (117), delivery by caesarean section (693), and instrumental vaginal delivery (144). Causes of exclusion of newborns included prematurity and low birth weight (70), presence of congenital anomalies and birth trauma (69), maternofetal incompatibility (22), and family history of hemolytic anemia (47). 

An informed consent was taken from each mother before enrollment in the study. 

### 2.1. Clinical Evaluation

Detailed history was obtained from each woman including parity and symptoms suggestive of VAD: recurrent urinary and respiratory infections and symptoms of dry eye (eye discomfort, eye dryness, foreign body sensation, photophobia, and night blindness). Mothers were asked to recall everything consumed (including foods and fluids) a day representing their usual intake starting from first meal or beverage on awakening until midnight of the reporting day. Data were analyzed and vitamin A intake was calculated using the “Diet Analysis Program, 1995” (Lifestyles Technologies, Inc., Northbridge Point, Valencia, CA). Physical Examination was done with stress on eye examination for dry eyes using 3 simple noninvasive tests. The tests were carried out in sequence, starting with tear film break-up time (TBUT), followed by examination of the cornea by fluorescein staining and the Schirmer I test without topical anesthesia. The Schirmer strips (Tianjin Jingming New Technological Development Co., Ltd., China) were inserted into the lower conjunctival sac at the junction of the lateral and middle thirds, avoiding touching the cornea, and the length of wetting strips in millimeters was recorded after 5 minutes. The cutoff point used for diagnosis of dry eye was <10 mm per 5 minutes [11].

For the newborns, Apgar scores were recorded at 1 and 5 minutes to exclude the presence of perinatal asphyxia [12]. Birth weight was measured by a digital baby scale. Length and occipitofrontal circumference (OFC) were measured by the same investigator. Gestational age was estimated using the new Ballard scoring system [13]. Systemic examination was done to exclude hepatosplenomegaly and congenital anomalies.

### 2.2. Laboratory Investigations

Five mL of maternal blood was collected by venipuncture immediately before delivery of the newborn. Cord blood was collected at delivery from the placental end of the cord; about 5 mL of mixed arterial and venous blood were collected. Each of the maternal and cord blood samples was divided into two specimens. One specimen was collected on EDTA tube for CBC (for both mothers and newborns), reticulocyte count and Coombs' test (for newborns only). According to WHO, maternal anemia was considered when Hb% is below 11 gm% [5]. The other specimen was collected in an autoclaved glass vial for serum retinol concentration measurement. The vials were immediately wrapped in aluminum foil to avoid photooxidation of vitamin A, were stored at 4°C, and were allowed to clot. After centrifuging the blood samples, the serum was carefully pippeted off into another vial and stored in a dark container at −20°C until analysis. Measurement of serum retinol concentration was done by high-performance liquid chromatography (HPLC) using reversed-phase column and diode-array detectors [14]. According to the WHO, we used maternal serum retinol level ≤0.7 *μ*mol/L as a cutoff value for maternal VAD [2].

### 2.3. Statistical Analyses

The data were coded and analyzed with the Statistical Package for Social Sciences (version 17; SPSS Inc, Chicago, IL, USA). Description of quantitative variables was presented as mean and SD, and that of categorical variables was presented as frequency and percentage. Unpaired *t*-test was used to compare parametric quantitative variables between the 2 groups: mothers with VAD and mothers without VAD. Chi square (*χ* ^2^) test was used to compare categorical variables between both groups. Pearson's correlation test was used for correlating maternal serum retinol concentrations with different variables. For all analyses, the level of significance was set at *P* value <0.05.

## 3. Results 

The age of the included ranged between 19 and 37 years with a mean of 26.4 ± 3.9 years. Four mothers (2%) gave history of night blindness and had signs of dry eyes. The maternal retinol intake ranged between 217.7 and 1300 *μ*g/day with a mean of 943.4 ± 433.1 *μ*g/day and a median of 435.5 *μ*g/day (390–890). One hundred and forty-one mothers (70.5%) had retinol intake ≤800 ug/day. 

The mean maternal Hb% was 10.7 ± 1.2 g% with a range between 6.6 and 13 g% and 50% of the mothers were anemic with a mean Hb% of 9.6 ± 1 gm% and 50% were nonanemic with a mean Hb% of 11.6 ± 0.4 gm%. Anemic mothers had a lower mean serum retinol concentration (1.2 ± 0.7 *μ*mol/L) compared to nonanemic mothers (1.9 ± 0.7), but the difference was not statistically significant, *P* = 0.07. 

The maternal serum retinol concentrations ranged between 0.31 and 3.6 *μ*mol/L with a mean of 1.6 ± 0.8 *μ*mol/L. Forty-seven mothers (23.5%) had VAD with a mean serum retinol concentration of 0.56 ± 0.14 *μ*mol/L. Mothers with VAD had a significantly lower mean retinol intake compared to mothers without VAD, with a significant positive correlation between maternal serum retinol concentration and retinol intake (*r* = 0.44 and *P* = 0.001). No significant differences were found between both groups regarding age and parity (Table 1). 

**Figure 1:**
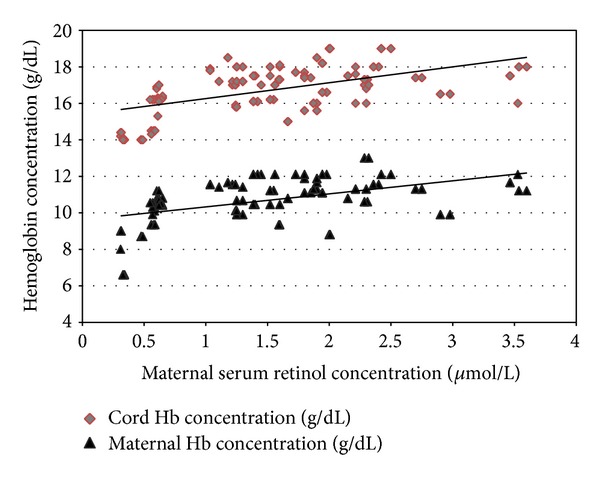
Correlations between maternal serum retinol concentrations and maternal and cord hemoglobin concentrations. Pearson's correlation test was used for correlating maternal serum retinol concentrations with maternal Hb% (*r* = 0.487 and *P* = 0.001) and cord Hb% (*r* = 0.531 and *P* = 0.001).

Mothers with VAD had a significantly lower mean Hb% (8.95 ± 1.63 gm%) compared to mothers without VAD (10.11 ± 0.83 gm%), *P* = 0.007, with a significant positive correlation between maternal serum retinol concentrations and maternal Hb% (*r* = 0.487 and *P* = 0.001) (Figure 1). Mothers with VAD had a significantly higher frequency of anemia (95.7%) compared to mothers without VAD (35.9%), *P* = 0.001.

The relative risk for anemia among mothers with VAD was 2.7 (CI = 2.12−3.3). The mean gestational age of included newborns was 38.1 ± 1.0 weeks with a range between 37 and 40 weeks. The mean birth weight was 3330 ± 307 gm with a range between 2890 and 3950 gm. The mean OFC was 34.5 ± 0.9 cm with a range between 33 and 36 cm. The mean length was 46.6 ± 0.9 cm with a range between 47 and 50 cm. The mean Hb% of all newborns was 16.8 ± 1.3 gm/dL with a range between 14.0 and 19.0 gm/dL. The mean level of cord serum retinol was 1 ± 0.45 *μ*mol/L with a range of 0.28 to 2.23 *μ*mol/L.

Newborns delivered to mothers with VAD had significantly lower mean values of Hb%, MCV. MCH and MCHC compared to newborns delivered to mothers without VAD (Table 2) with a significant positive correlation between maternal serum retinol concentrations and cord Hb% (*r* = 0.531 and *P* = 0.001) (Figure 1). No significant differences between the two groups regarding gestational age, anthropometric measurements, WBCs, and platelet counts of the newborns (Table 2).

Newborns delivered to mothers with VAD had a significantly lower mean cord serum retinol concentration (0.43 ± 0.1 *μ*mol/L) compared to newborns delivered to mothers without VAD (1.19 ± 0.42 *μ*mol/L). The cord serum retinol concentration of all newborns had a significantly positive correlation with the serum retinol concentration of their respective mothers (*r* = 0.952 and *P* < 0.001).

## 4. Discussion

In Egypt, VAD during pregnancy represents a major public health problem. In a recent study, El-Khashab et al. (2013) found that 20% of pregnant women had VAD [15]. In other developing countries, VAD was found among 15.8% (in Nigeria) and 18.8% (in Bangladesh) of pregnant women [16, 17]. The frequency of mothers with retinol intake below the recommended intake (70%) is higher than that reported from other developing countries (53%) [17]. The positive correlation between the maternal serum retinol concentrations and maternal vitamin A intake is documented in many previous studies [18]. The higher frequency of VAD in the present study may be explained by inclusion of only women of low income families.

The frequency of anemia among included pregnant females (50%) is similar to that reported from West and Central Africa [19] and other developing countries [6].

Significantly lower mean Hb% among pregnant women with VAD compared to healthy women and significant positive correlations between maternal serum retinol and maternal Hb% were reported in previous studies [20–22]. Women with VAD had 1.8 times greater risk of being anemic than did the women without VAD [23]. Vitamin A supplementation was found to improve hemoglobin concentrations [9] and reduces maternal anemia for women who live in areas where VAD is common [24]. The mechanisms of anemia resulting from VAD and how vitamin A supplementation can improve hemoglobin have not been elucidated. These mechanisms fall into three general categories. First, modulation of erythropoiesis as retinoic acid was found to stimulate erythropoietin gene transcription [25]. Vitamin A supplementation was found to increase the circulating erythropoietin level [26]. The second mechanism is the anti-infective role [27] as infection is associated with decreased serum iron levels, suppressed erythropoiesis, and lower hemoglobin concentration [28]. The third mechanism is modulation of iron metabolism. It has been suggested that vitamin A is required for the mobilization and utilization of iron for hemoglobin synthesis. Vitamin A maintains iron homeostasis by the modulation of liver hepcidin expression [29] and regulation of iron regulator protein-2 (IRP2) [30]. In cases of VAD, iron is trapped in the liver and spleen and is not effectively released for erythropoiesis by bone marrow [8].

The nonsignificant difference between anemic and non-anemic mothers regarding serum retinol concentrations indicates that VAD is not the only cause of anemia during pregnancy. Causes of anemia during pregnancy include iron deficiency (most common cause), other micronutrient deficiencies (zinc, copper, vitamin B_12_, and folic acid), hemoglobinopathies (sickle cell disease and thalassemia), and human pathogens in certain geographical populations such as hookworm, malaria, and human immunodeficiency virus [31, 32]. Because the present study aimed at correlating maternal VAD with maternal anemia and neonatal Hb% and the lack of financial support, we could not assess iron status for all included women. 

## 5. Conclusion

Maternal VAD during pregnancy is associated with maternal anemia and lower Hb% of the newborns at birth. Vitamin A supplementation during pregnancy is recommended especially in low income countries to decrease the frequency of anemia.

## Figures and Tables

**Table 1:** Comparisons between mothers with vitamin A deficiency and mothers without vitamin A deficiency regarding age, parity, vitamin A status, and hemoglobin concentration.

	Mothers with VAD *N* = 47	Mothers without VAD *N* = 153	*t* value	*P*
Maternal age (years)	26.0 ± 5.3	26.8 ± 3.4	1.072	0.29
Parity	1.9 ± 0.9	1.6 ± 1.1	0.926	0.36
^ 1^Retinol intake (*µ*g/day)	346.5 ± 81.9	673.9 ± 274.4	3.412	**0.002**
Serum retinol (*µ*mol/L)	0.5 ± 0.1	1.9 ± 0.6	−14.816	**0.001**
Hb% (g/dL)	9.5 ± 1.3	11.1 ± 0.9	−9.557	**0.001**

VAD: vitamin A deficiency; Hb%: hemoglobin concentration.

Data are presented as mean ± SD. Unpaired *t*-test was used for comparisons. *P* value < 0.05 indicates a significant difference.

^1^Retinol intake was measured by analysis of 24 hours dietary recall using “Diet Analysis Program, 1995” (Lifestyles Technologies, Inc., Northbridge Point, Valencia, CA).

**Table 2:** Comparisons between newborns of mothers with vitamin A deficiency and newborns of mothers without vitamin A deficiency regarding anthropometric measurements, hematological parameters, and cord serum retinol concentrations.

	Newborns of mothers with VAD *N* = 47	Newborns of mothers without VAD *N* = 153	*t* value	*P*
Gestational age (week)	38.2 ± 1.3	38.05 ± 0.9	0.936	0.351
Weight (kg)	3.37 ± 0.36	3.32 ± 0.29	1.033	0.303
OFC (cm)	34.3 ± 1.1	34.5 ± 0.9	1.228	0.227
Length (cm)	46.7 ± 0.82	46.5 ± 0.85	1.06	0.289
WBCs (×10^3^/mm^3^)	8.5 ± 1.4	8.6 ± 1.3	0.154	0.852
Hb% (g/dL)	15.3 ± 1.1	17.2 ± 0.9	−11.6	**<0.001**
MCV (fL)	95.9 ± 2.3	97.5 ± 2.7	−3.42	**0.001**
MCH (pg)	32.6 ± 1.3	33.2 ± 1.1	−2.649	**0.009**
MCHC	33.8 ± 1.1	34.6 ± 0.7	−5.788	**<0.001**
Platelets (×10^3^/mm^3^)	275.7 ± 53.6	259.6 ± 86.4	1.9	0.08
Cord retinol concentration (*µ*mol/L)	0.43 ± 0.1	1.2 ± 0.4	−13.313	**0.001**

VAD: vitamin A deficiency; OFC: occipitofrontal circumference; WBCs: white blood cells; Hb%: hemoglobin concentration; MCV: mean corpuscular volume; MCH: mean corpuscular hemoglobin; MCHC: mean corpuscular hemoglobin concentration.

Data are presented as mean ± SD. Unpaired *t*-test was used for comparisons. *P* value < 0.05 indicates a significant difference.

## Abbreviations

VAD: Vitamin A deficiency
Hb%: Hemoglobin concentration
OFC: Occipitofrontal circumference
TBUT: Tear film break-up time
MCV: Mean corpuscular volume
MCH: Mean corpuscular hemoglobin
MCHC: Mean corpuscular hemoglobin concentration
IRP2: Iron regulator protein-2.
